# Extracellular Adenosine Diphosphate Stimulates CXCL10-Mediated Mast Cell Infiltration Through P2Y1 Receptor to Aggravate Airway Inflammation in Asthmatic Mice

**DOI:** 10.3389/fmolb.2021.621963

**Published:** 2021-07-05

**Authors:** Yan-Yan Gao, Zeng-Yan Gao

**Affiliations:** Department of Respiratory Medicine, The Affiliated Hospital of Weifang Medical University, Weifang, China

**Keywords:** adenosine diphosphate, ovalbumin, asthma, airway inflammation, mast cells, C-X-C motif chemokine ligand 10, NF-κB signaling pathway

## Abstract

Asthma is an inflammatory disease associated with variable airflow obstruction and airway inflammation. This study aimed to explore the role and mechanism of extracellular adenosine diphosphate (ADP) in the occurrence of airway inflammation in asthma. The expression of ADP in broncho-alveolar lavage fluid (BALF) of asthmatic patients was determined by enzyme linked immunosorbent assay (ELISA) and the expression of P2Y1 receptor in lung tissues was determined by reverse transcription-quantitative polymerase chain reaction. Asthmatic mouse model was induced using ovalbumin and the mice were treated with ADP to assess its effects on the airway inflammation and infiltration of mast cells (MCs). Additionally, alveolar epithelial cells were stimulated with ADP, and the levels of interleukin-13 (IL-13) and C-X-C motif chemokine ligand 10 (CXCL10) were measured by ELISA. We finally analyzed involvement of NF-κB signaling pathway in the release of CXCL10 in ADP-stimulated alveolar epithelial cells. The extracellular ADP was enriched in BALF of asthmatic patients, and P2Y1 receptor is highly expressed in lung tissues of asthmatic patients. In the OVA-induced asthma model, extracellular ADP aggravated airway inflammation and induced MC infiltration. Furthermore, ADP stimulated alveolar epithelial cells to secrete chemokine CXCL10 by activating P2Y1 receptor, whereby promoting asthma airway inflammation. Additionally, ADP activated the NF-κB signaling pathway to promote CXCL10 release. As a “danger signal” extracellular ADP could trigger and maintain airway inflammation in asthma by activating P2Y1 receptor. This study highlights the extracellular ADP as a promising anti-inflammatory target for the treatment of asthma.

## Introduction

Asthma has been recognized as one of the common non-communicable chronic diseases in children and adults, clinically manifested with airway inflammation and remodeling (Christiansen and Zuraw, 2019). Notably, approximately 250,000 asthma-related deaths occur each year, many of which are avoidable (Barcik et al., 2020). The presence of airway inflammation in asthma is commonly induced by either viruses or allergen and related to secretion of inflammatory cytokines from epithelia cells and a diversity of lung resident cells such as mast cells (MCs), and macrophages, as well as recruited cells including eosinophils, T cells, and neutrophils (Chen et al., 2018; Nakagome and Nagata, 2018). A previous study has also demonstrated that activation of MCs along with infiltration and activation of eosinophils and T cells are implicated in airway inflammation of asthmatic patients (Barnes, 2018). Recently, anti-inflammatory and bronchodilator therapies are regarded as the main treatment methods for asthma (Papi et al., 2018). Therefore, exploring the mechanism underlying the pathogenesis of airway inflammation in asthma may lay a theoretical foundation for the development of novel targeted therapies.

Extracellular adenosine diphosphate (ADP), as a kind of purinergic signals, is capable of regulating communications among cells and stress responses in majority of mammalian tissues (Trull et al., 2019). ADP is a critical regulator of cell viability, stress response, immunity, membrane permeability, and growth (Demidchik et al., 2011). Purinergic receptors also involve in airway defense mechanisms, while extracellular purines such as ADP can be used as new biomarkers of airway inflammation in asthma (Di Virgilio and Adinolfi, 2017; Trinh et al., 2019). Interestingly, P2Y1 receptor is a member of the family of G protein-coupled receptors which are implicated in the pathogenesis of allergic asthma such as platelet activation and aggregation, and inflammatory processes while ADP is known as a physiological agonist of these receptors (Rafehi and Muller, 2018; Gachet and Hechler, 2020). C-X-C motif chemokine ligand 10 (CXCL10) is a chemokine induced by interferon-γ that is associated with a series of chronic inflammatory disorders (Aota et al., 2018). Furthermore, the antagonist of either P2Y1 receptor or P2Y12 receptor can reduce the expression of CXCL10 (Kuboyama et al., 2011; Halim et al., 2019). Most importantly, CXCL10 is a MC attractant released from airway smooth muscle cells (ASMCs) that exerts a crucial role in the accumulation of MCs in airway smooth muscle (Seidel et al., 2012). According to the above findings, it is hypothesized that extracellular ADP may mediate CXCL10 through P2Y1 receptor to affect the infiltration of MC and the airway inflammatory response in asthma. In the present study, we sought to test this hypothesis and to identify the interaction between these factors in the pathogenesis of airway inflammation in asthma.

## Materials and Methods

### Ethics Statement

Written informed consents were obtained from all participants. The experiment involved human beings was approved by the Ethics Committee of The Affiliated Hospital of Weifang Medical University. All animal experiments in this study were approved by the Animal Ethics Committee of The Affiliated Hospital of Weifang Medical University and in accordance with the principles of local laboratory animal management and use.

### *In silico* Analysis

Based on existing literature and asthma-related gene expression microarray GSE64913 obtained from Gene Expression Omnibus (GEO) database (https://www.ncbi.nlm.nih.gov/gds), the key ADP-mediated P2Y receptors and downstream genes were predicted. The microarray GSE64913 included 42 normal samples and 28 asthma samples. The differentially expressed genes were analyzed using the “limma” package of R language (https://bioconductor.org/packages/limma/), with *p* < 0.05 as the threshold. String (https://string-db.org/) was used to predict 10 genes which interacted with predicted downstream genes. A protein-protein interaction network was plotted through Cytoscape (https://cytoscape.org/), KOBAS (https://bioconductor.org/packages/limma/ was employed to conduct gene ontology (GO) and Kyoto encyclopedia of genes and genomes (KEGG) enrichment analysis of downstream genes and 10 interaction genes to determine the main functions of downstream genes.

### Sample Collection

Twenty asthmatic patients (36.2 ± 2.2 years old) who received systemic treatment in The Affiliated Hospital of Weifang Medical University from June 2017 to May 2019 were selected as experimental subjects, including 12 males and eight females. At the same time, 15 healthy volunteers (33.21 ± 4.5 years old) who had no history of asthma or other chronic lung diseases were selected as the control group, including nine males and six females. Asthma was diagnosed by at least one positive objective diagnostic test such as methacholine test (PD20 < 8 mmol), mannitol test (PD15 < 635 mg), FEV1 reversibility to a b2-agonist of 12% or ≥ 250 ml. The patients had asthmatic symptoms in the last four weeks. The patients with smoking history within at least the past six months, medicine history of no corticosteroids, or other chronic lung diseases were excluded.

The broncho-alveolar lavage fluid (BALF) from the upper left lobe of the lung was collected and subjected to lavage with 2 × 60 ml saline, followed by centrifugation at 4,000 rpm for 10 min. The supernatant was taken for further analysis and testing. The biopsy tissues collected from the right middle and lower lobe of the lung were immediately immersed in 4% formaldehyde, and then embedded in paraffin.

### Extracellular Adenosine Diphosphate Concentration Detection

The operation was performed according to the instructions of the ADP detection kit (Sigma, St. Louis, MO, United States), and the reaction solution was prepared according to the Table 1. The obtained BALF was reacted with the reacted reaction solution at room temperature in the dark for 30 min. The optical density (OD) value at 570 nm (A570) was measured. The standard curve was drawn according to the relationship between the amount of standard and the OD value, and then the amount of ADP was calculated corresponding to the OD value of the sample found on the standard curve using the formula in the Table 1.

**TABLE 1:** The reaction solution for ADP detection.

Reagent	Blank control	Sample and standard (μL)
ADP Assay buffer	46 μl	46
ADP probe	2 μl	2
ADP converter	—	2
ADP developer	2 μl	2

### Ovalbumin-induced Asthmatic Mouse Model Construction

The wild-type (WT) C57BL/6 male mice (6–8 weeks) were purchased from Shanghai Lab. Animal Research Center. All mice were housed under specific pathogen free (SPF) conditions and reared according to institutional guidelines. According to the experimental requirements, the asthma model was induced using OVA. In detail, 100 μg of OVA (grade V, Sigma) was mixed with 1% aluminum hydroxide solution (Sigma) in phosphate buffered saline (PBS). Then, 200 μL of emulsified OVA was intraperitoneally injected into wild-type mice, which was repeatedly on the 0th, 7th, and 14th days. On days 21–27, the sensitized mice were stimulated with aerosolized OVA (1%) for 30 min each time. Twenty-four h after the last atomization, the mice were anesthetized with 5% avertin before the experiment, and then all mice were euthanized by cervical dislocation, with the serum, BALF and lung tissues collected (Casaro et al., 2019).

The mice were grouped into a PBS/PBS/OVA group (sensitization with PBS + nasal drops with PBS + atomization with OVA), an OVA/PBS/OVA group (sensitization with OVA + nose drops with PBS + atomization with OVA), an OVA/apyrase/OVA group (sensitization with OVA + nose drops with apyrase + atomization with OVA), and an OVA/apyrase + ADP/OVA group (sensitization with OVA + nose drops with apyrase/ADP + atomization with OVA).

Additionally, the WT mice received sensitization with OVA + nasal drops with PBS + atomization with OVA, or sensitization with OVA + nasal drops with ADP + atomization with OVA. The P2Y1 knockout (KO) mice received sensitization with OVA + nasal drops with PBS + atomization with OVA, or sensitization with OVA + nose drops with ADP + atomization with OVA.

### Analyses of Cytokines in BALF

The lungs were rinsed twice in 1 ml PBS containing 0.5% fetal bovine serum (FBS). The BALF was obtained after lavage and centrifuged at 2000 g for 5 min at 4°C. The sediment was resuspended in 50 μL PBS, and the total number of cells was counted with a hemocytometer. After the supernatant was collected, the protein levels of IL-13 and CXCL10 in the BALF were detected using the corresponding enzyme linked immunosorbent assay (ELISA) kit according to the manufacturer’s instructions.

### Flow Cytometry

Cells were harvested from the BALF, and washed with Cell Staining Buffer (420,201, Dakewe Biotech Co., Ltd., Shenzhen, Guangdong, China). The cells were incubated with anti-CD117 PE (MC) (BioLegend, San Diego, CA, United States) in 50 μL staining buffer for 30 min. The cells were washed twice with staining buffer and resuspended in 200 μL staining buffer. The distribution of immune cells in BALF was analyzed on the FACSCalibur (BD Biosciences, San Jose, CA, United States).

The lung tissues were cut into millet grains-like fragments and rinsed in pre-cooled Hank’s Balanced Salt Solution (HBSS) for several times. The fragments were placed in a culture flask, which was placed in an incubator at 37°C. After 4 h of digestion, the fragments were cultured with new medium for another 24 h. The lung tissue fragments were observed under the microscope to analyze the status of cells, and then the medium was changed. Finally, the proportion of mast cells (MCs) was analyzed on the FACSCalibur (BD Biosciences).

### Histological Analysis

The left upper lobe of lung in each mouse was fixed with 4% paraformaldehyde for 24 h, embedded with paraffin, and sliced into 5-μm-thick sections. Briefly, sections were deparaffinized in xylene and immersed separately in 100, 90, 70 and 50% ethanol for gradient rehydration. After staining with hematoxylin and eosin, the sections were gradually dehydrated and mounted, after which the pulmonary inflammation was observed with an optical microscope.

The sections were incubated in periodic acid ethanol before staining with Periodic Acid Schiff (PAS). The sections were washed with sulfurous acid, and the nuclei were stained with hematoxylin and mounted with glycerol. The PAS-positive goblet cells and mucus secretion were observed under an optical microscope.

### Cell Culture

The cryo-preserved alveolar epithelial cells (SW1573) and MCs (HMC-1) were quickly thawed in a 37°C water bath to thaw. After the cells were completely thawed, the culture solution in the cryopreservation tube was transferred to a 15 ml centrifuge tube, and centrifuged at 1,200 rpm for 5 min. The obtained cells were precipitated and resuspended in 5 ml complete culture medium [Dulbecco’s modified Eagle’s medium (DMEM) + 10% FBS +1% PBS], which were transferred to T25 cell culture flask, and placed in a 5% CO_2_ incubator at 37°C. The cell growth was observed on time, and the passage could be started when the cell confluence reached about 80%. After original culture medium was discarded, the cells were washed twice with PBS, and detached with 1 ml of trypsin. When cells were observed to be round-shaped with bright edges under an inverted microscope, the trypsin was sucked away. After addition of 2 ml fresh culture medium, the cells were gently dispersed into cell suspension. 1 mlcell suspension was added into new bottle and cultured with 5 ml of culture medium in the incubator at 37°C with 5% CO_2_.

### Transwell Assay

After the cells were seeded in a 6-well culture plate at a density of 1 × 10^5^ cells/well and cultured overnight, the serum-free DMEM was replaced. The cells were stimulated with 100 μM ADP or an equal volume of PBS for 12 h, and the cell culture supernatant was harvested for use. The ADP-treated cell culture supernatant was loaded into the 24-well plate (1 ml/well) which was added with 3 μm transwell chamber for hydration. The serum-free medium was used as a negative control, and the medium containing 2% FBS as a positive control. The MCs were seeded to the chamber (1 × 10^5^ cells/well), and placed in the incubator at 37°C for 5 h. The chambers were removed, and the medium in the well was transferred to a centrifuge tube, and centrifuged at 1,500 rpm for 5 min. The cells were resuspended in fluorescence activated cell sorting (FACS) staining buffer and then added to the flow tube. The number of migrated cells was analyzed on the FACSCalibur (BD Biosciences) and the chemotactic index was calculated.

### Immunohistochemical Staining

The paraffin-embedded sections were dewaxed and rehydrated. The sections were placed in the pre-boiled 1× antigen retrieval, boiled at 100°C for 30 min, and then cooled down to room temperature. The slices were immersed in a 3% hydrogen peroxide for 5 min to block the endogenous peroxidase activity, and permeabilized in a solution containing 0.1 Triton X-100 for 5 min. After washing twice with PBS, the slices were incubated with goat serum at room temperature for 20 min for blocking, and then incubated with diluted primary antibody overnight at 4°C in a humidified box. Next day, the primary antibody on the slices was discarded. The sections were washed twice with PBS, and incubated with fluorescence-labeled second antibody at room temperature for 1 h. After washing twice with PBS, the sections were mounted with anti-fluorescence quenching agent, observed and photographed with a fluorescence microscope.

### Reverse Transcription-Quantitative Polymerase Chain Reaction

Total RNA was extracted by TRIzol reagents (15596026, Invitrogen, Carlsbad, CA, United States), and then reversely transcribed into cDNA according to the instructions of PrimeScript RT reagent Kit (RR047A, Takara, Tokyo, and Japan). The synthesized cDNA was subjected to RT-qPCR using Fast SYBR Green PCR kit (Applied Biosystems, Foster City, CA, United States) and ABI^PRISM^ 7300 RT-PCR system (Applied biosystems), with three replicates in each well. Glyceraldehyde-3-phosphate dehydrogenase (GAPDH) was used as an internal reference and the 2^−ΔΔCt^ method was used to analyze the relative expression of targets. ΔΔCt = (ΔCt _target gene in the experimental group_–ΔCt _housekeeping gene in the experimental group_)–(ΔCt _target gene in the control group_–ΔCt _housekeeping gene in the control group_). The primer sequences are shown in Table 2.

**TABLE 2:** Primer sequences of RT-qPCR.

Gene	Primer sequence (5′-3′)
CXCL10	Forward: 5′-GTG​GCA​TTC​AAG​GAG​TAC​CTC-3′
	Reverse: 5′-TGA​TGG​CCT​TCG​ATT​CTG​GAT​T-3′
GAPDH	Forward: 5′-GTG​GAC​CTG​ACC​TGC​CGT​CT-3′
	Reverse: 5′-GGA​GGA​GTG​GGT​GTC​GCT​GT-3′
hP2RY12	Forward: 5′-ATG​CGC​CTT​GAC​CAA​GAC​G-3′
	Reverse: 5′-GGC​CAG​AGC​CAA​ATT​GAA​CA-3′

Note: RT-qPCR, reverse transcription-quantitative polymerase chain reaction; CXCL10, C-X-C motif chemokine ligand 10; GAPDH, glyceraldehyde-3-phosphate dehydrogenase.

### Western Blot Analysis

SW1573 cells were seeded into a 6-well plate at a density of 1 × 10^6^ cells/well, and after overnight culture, the cells were treated with 100 μM ADP or inhibitor according to the time and conditions required by the experiment. After the culture medium was discarded, the cells were washed with pre-cooled PBS, and lysed with 300 μL radioimmunoprecipitation assay (RIPA) lysis buffer containing phosphatase inhibitor or protease inhibitor on ice for 30 min. The cells were centrifuged at 12,000 rpm and 4°C for 5 min, and then the supernatant was collected and stored at−80°C. The protein was diluted with 5 × loading buffer, and boiled in a metal bath at 100°C (20 μL/well) for 10 min. The protein was loaded on protein vertical electrophoresis device. The concentrated gel was electrophoresed at a constant voltage of 60 V for 1 h, and the separation gel was electrophoresed at a constant voltage of 80 V for 2–3 h. After the separated proteins were transferred onto membranes at a constant current of 350 mA for 1 h, the membrane was incubated with the primary antibody (1: 1,000) overnight at 4°C. The membrane was washed with PBST for three times, 10 min each time, and incubated with secondary antibody (1: 7,500) for 1 h at room temperature. The membrane was washed again with PBST for three times, 10 min each time, and scanned with Odyssey fluorescent membrane scanner.

### Statistical Analysis

SPSS 21.0 statistical software (IBM Corp, Armonk, NY, United States) was used for the statistical analysis. Measurement data were expressed as mean ± standard deviation. Unpaired *t* test was used for comparison between two variables, while one-way analysis of variance (ANOVA) was used for data comparison among multiple groups followed by Tukey’s post hoc test. *p* < 0.05 means the difference was statistically significant.

## Results

### Potential Significance of P2Y1 and CXCL10 in Asthma

**FIGURE 1:**
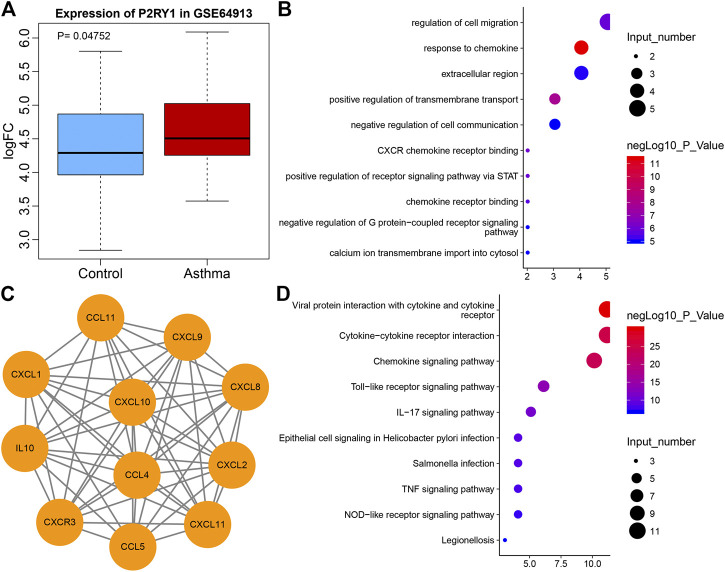
The potential significance of P2Y1 and CXCL10 in asthma. **(A)** Box plot of P2Y1 expression in normal samples (blue) and asthma samples (red) in microarray GSE64913. **(B)** 10 genes that interacted with CXCL10 predicted by String. **(C)** The GO enrichment map of CXCL10 and 10 interaction genes. The vertical axis represents the enriched entry, and the horizontal axis represents the number of genes enriched in the entry. The color of the bubble as shown by the color scale on the right indicates the enrichment significance in the entry. **(D)** The KEGG enrichment map of CXCL10 and 10 interaction genes, the vertical axis represents the enriched item, the horizontal axis represents the number of genes enriched in the item, and the color of the bubble indicates the enrichment significance as shown in the color scale on the right.

Extracellular ADP has been reported to regulate P2 receptor to affect inflammation (Feldbrugge et al., 2020). ADP-related P2 receptors include P2Y1, P2Y12, and P2Y13 (Hockley et al., 2016). According to the differential analysis of microarray GSE64913, P2Y1 was found to be differentially expressed in asthma (Figure 1A), and P2Y1 is also listed as P2RY1 in National Center for Biotechnology Information (NCBI). A recent study has demonstrated that ADP can activate P2Y1 (Cacciari et al., 2019), while another study has suggested that P2Y1 can promote the expression of CXCL10 (Kuboyama et al., 2011). To explore the mechanism of P2Y1 in asthma through CXCL10, String was used to predict the 10 genes that interacted with CXCL10 and a protein interaction network was plotted (Figure 1B). GO and KEGG enrichment analysis of these 11 genes were conducted using KOBAS and results revealed that the main GO entries included response to chemokine, positive regulation of transmembrane transport, CXCR chemokine receptor binding, positive regulation of receptor signaling pathway *via* STAT, and regulation of cell migration (Figure 1C). The main KEGG entries included viral protein interaction with cytokine and cytokine receptor, cytokine-cytokine receptor interaction, chemokine signaling pathway, toll-like receptor signaling pathway and IL-17 signaling pathway (Figure 1D). Those entries correlated with inflammation. Furthermore, CXCL10 overexpression can induce infiltration of MCs to aggravate asthma (Gao et al., 2014; Gauthier et al., 2017). Hence, it was speculated that extracellular ADP can regulate CXCL10-mediated infiltration of MCs through P2Y1, thereby aggravating airway inflammation of asthmatic patients.

### High Concentrations of Extracellular ADP in the BALF of Asthmatic Patients

**FIGURE 2:**
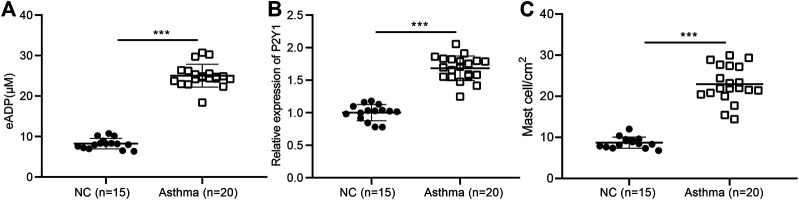
High concentrations of extracellular ADP in the BALF of asthmatic patients. **(A)** ADP release in BALF of asthma patients (*n* = 20) and healthy volunteers (*n* = 15). ***p < 0.001 compared with the healthy volunteers. **(B)** P2Y1 receptor expression in lung tissues of asthma patients (*n* = 20) and healthy volunteers determined by RT-qPCR (*n* = 15). **(C)** Toluidine blue staining of the infiltration of MCs in the lung tissue of asthma patients. ***p < 0.001 compared with the healthy volunteers or PBS treatment. Measurement data were expressed by the mean ± standard deviation, analyzed by independent sample *t* test, and the experiment was repeated three times.

Studies have shown that ADP, as an extracellular “danger signal”, played an important role in the occurrence of inflammation (Cattaneo, 2015; Suzuki et al., 2020), but its role in airway inflammation in asthma has not been studied. The BALF was collected from 20 asthma patients and 15 healthy volunteers, and the result showed that the level of extracellular ADP in the BALF of asthma patients was significantly higher than that in the healthy volunteers (Figure 2A). At the same time, the results of RT-qPCR revealed that the ADP receptor P2Y1 was highly expressed in the lung tissues of asthma patients. Consistently, P2Y1 was expressed at higher level in lung tissues of asthma patients than in lung tissues of healthy volunteers (Figure 2B). Toluidine blue staining analysis suggested that the inflammation was severe in the lung tissue of asthma patients (Figure 2C). Hence, ADP and its receptor P2Y1 may play an important regulatory role in the pathogenesis of asthma.

### Extracellular ADP Aggravates Airway Inflammation in Asthmatic Mice

**FIGURE 3:**
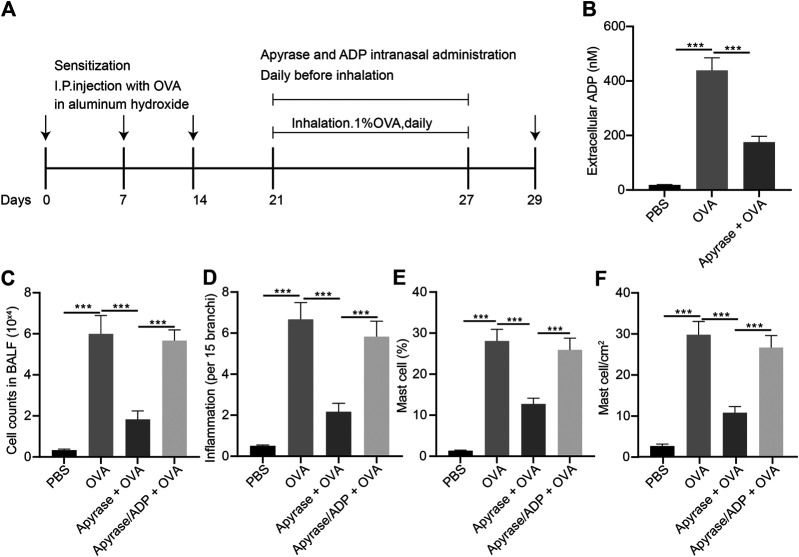
Extracellular ADP aggravates OVA-induced airway inflammation in asthmatic mice. **(A)** Schematic diagram of OVA-induced asthmatic mouse model construction and drug treatment process. **(B)** Quantitative analysis of ADP levels in BALF of mouse. **(C)** The number of infiltrated various types of immune cells the lungs of mice analyzed by FCM. **(D)** Quantitative analysis of pathological scores of lung inflammation assessed by HE staining. **(E)** Proportion of MCs infiltrated in the lungs of mice analyzed by FCM. **(F)** Distribution of MCs in the lungs evaluated by toluidine blue staining. *p < 0.05, **p < 0.01, and ***p < 0.001. Measurement data were expressed by the mean ± standard deviation, analyzed by independent sample *t* test, and the experiment was repeated three times.

In order to study the effect of extracellular ADP on airway inflammation in asthma, a mouse model of asthma induced by OVA was constructed, and the mice were injected with apyrase enzyme and ADP intranasally before daily atomization of OVA (Figure 3A). Compared with PBS-induced mice, the ADP concentration of BALF was increased significantly in the mice sensitized by OVA before OVA atomization, while the release of ADP was decreased remarkably after treatment with apyrase (Figure 3B). Next, FCM was used to analyze all the inflammatory cells in the BALF of OVA-induced asthmatic mouse. The results presented that the number of inflammatory cell infiltration was notably reduced after intranasal injection of apyrase enzyme. After endogenous ADP was cleared by apyrase enzyme, mice were injected with exogenous ADP intranasally; as a consequence, the number of inflammatory cell infiltration was increased (Figure 3C). By analyzing the results of HE staining of pathological lung tissue sections of mice, it was found that lung inflammation in asthmatic mice could be alleviated by treatment with apyrase enzyme, while the lung inflammation caused by OVA was increased after supplementation of exogenous ADP (Figure 3D).

It was speculated that ADP was related to MC infiltration in the OVA-induced asthmatic mouse model. The infiltration of MCs was analyzed by FCM, the results of which showed that MC infiltration was significantly increased in the OVA-induced asthmatic mice, which was dramatically reduced after intranasal injection of apyrase enzyme. After the endogenous ADP was cleared by apyrase enzyme, the intranasal injection of exogenous ADP increased the number of MC infiltration (Figure 3E). As shown by toluidine blue staining in the lung tissue sections, the distribution of MCs was consistent with the results of FCM (Figure 3F). The aforementioned experimental results preliminarily demonstrate that ADP in the lungs of asthmatic mice may be involved in aggravation and regulation of inflammation, which can be effectively alleviated by clearing ADP.

### P2Y1 Involves in ADP-Mediated Airway Inflammation in Asthmatic Mice

**FIGURE 4:**
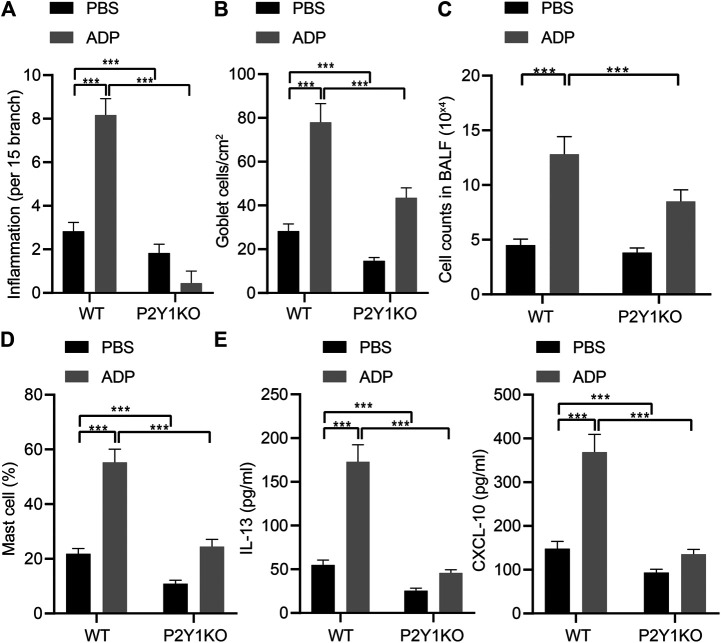
P2Y1 may be involved in ADP-mediated airway inflammation in asthmatic mice. **(A)** Quantitative analysis of pathological scores of lung inflammation. **(B)** Quantitative analysis of the number of PAS-positive goblet cells in the lungs. **(C)** The number of cells infiltrated in the lungs of mice. **(D)** The proportion of MCs infiltrating the lungs of mice. **(E)** The levels of cytokines IL-13 and CXCL10 in BALF of mice. *p < 0.05, **p < 0.01, and ***p < 0.001. Measurement data were expressed by the mean ± standard deviation, analyzed by independent sample *t* test, and the experiment was repeated 3 times.

In order to examine the function of ADP and its receptor P2Y1 in the occurrence of airway inflammation in asthma, CRISPR-Cas9 technology was used to construct P2Y1 gene knockout mice and wild-type and P2Y1-knockout mice were used to construct OVA-induced asthma model. Before being atomized by OVA, ADP or an equivalent amount of control PBS was nasally administered to the mice at a dose of 20 mg/kg. As a result, the degree of inflammation and goblet cell hyperplasia in the lung bronchi and blood vessels of wild-type mice after ADP administration was increased compared with P2Y1-knockout mice. In addition, the bronchial inflammation on lung and goblet cell hyperplasia of both wild-type and P2Y1-knockout mice were elevated by ADP treatment. The quantified pathological scores (Figure 4A) and the number of goblet cell positive staining cells (Figure 4B) were observed to be consistent. At the same time, the cells in BALF were counted, and the results revealed that the cell infiltration in lungs of wild-type mice was increased after ADP treatment, but that was sharply reduced in P2Y1-knockout mice (Figure 4C). Meanwhile, the results of FCM showed that changes in the proportion of MCs in the BALF of mice were consistent with these in total infiltrated cells (Figure 4D). After stimulation by ADP, the levels of cytokines were tested by ELISA, and it was found that compared with wild-type mice, the levels of Th2 cytokine IL-13 and chemokine CXCL10 were markedly reduced in P2Y1-knockout mice (Figure 4E). The above experimental results indicate that extracellular ADP may aggravate the inflammation in the lungs of asthmatic mice through the P2Y1 receptor.

### ADP Promotes the Expression of CXCL10 Through P2Y1 Receptor

**FIGURE 5:**
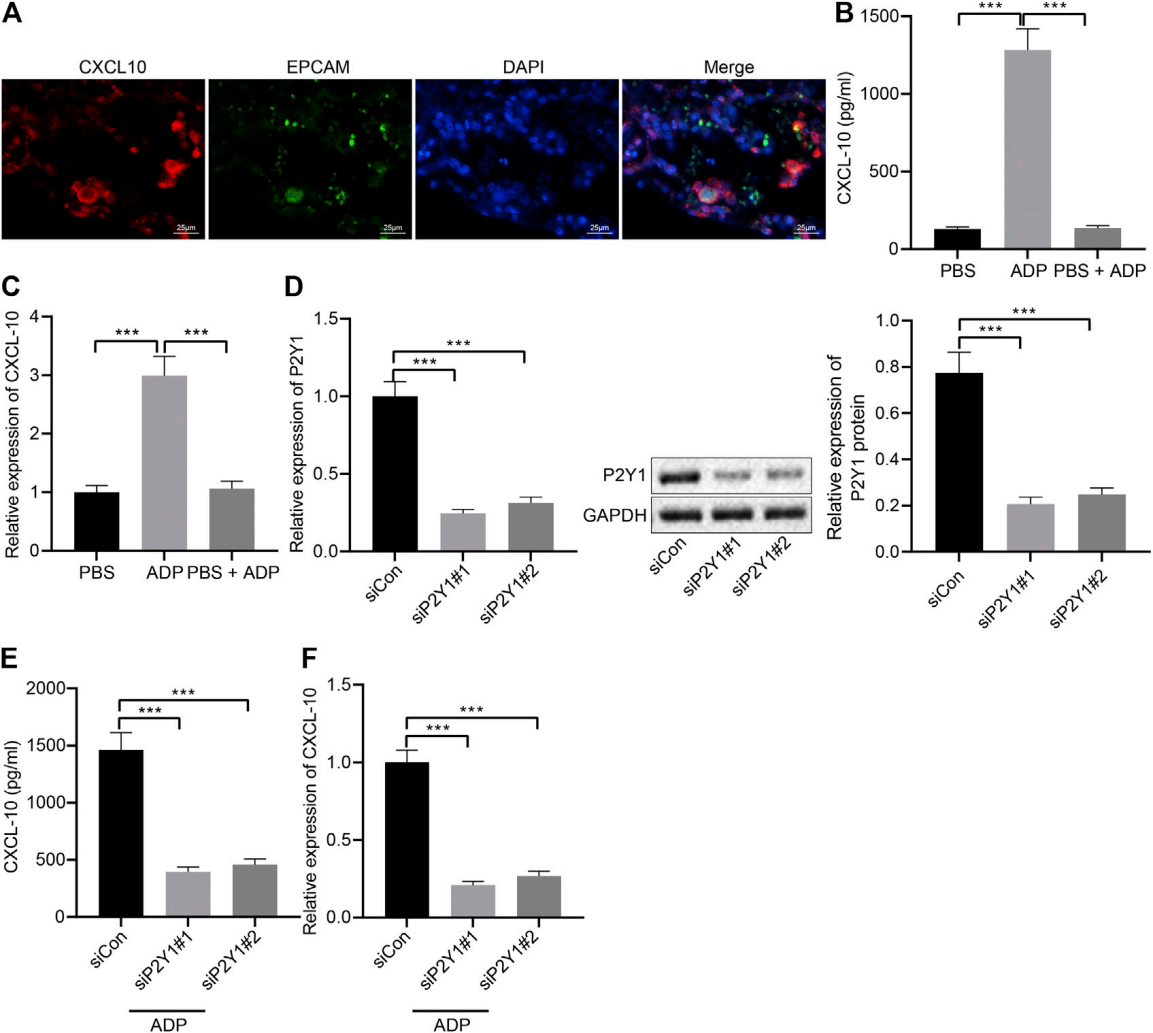
ADP promotes release of CXCL10 through P2Y1 receptor. **(A)** Representative microscopic images of immunofluorescence staining in lung tissues of asthmatic mice. The co-localization of alveolar epithelial cell markers EPCAM and CDH1 (green) and chemokine CXCL10 (red) was observed, and the nucleus was labeled with DAPI (blue). **(B)** The release of CXCL10 in alveolar epithelial cells SW1573 after P2Y1 receptor inhibition by PSB0739 (1 h) and ADP treatment (12 h). **(C)** The expression of CXCL10 in alveolar epithelial cells after P2Y1 receptor inhibition by PSB0739 (1 h) and ADP treatment (12 h) determined by RT-qPCR. **(D)** Knockdown efficiency of P2Y1-specific siRNA determined by RT-qPCR and Western blot analysis. **(E)** CXCL10 release in alveolar epithelial cells SW1573 after P2Y1 knockdown by specific siRNA and ADP treatment. **(F)** CXCL10 expression in alveolar epithelial cells SW1573 after P2Y1 knockdown by specific siRNA and ADP treatment determined by RT-qPCR. *p < 0.05, **p < 0.01, and ***p < 0.001. Measurement data were expressed by the mean ± standard deviation, analyzed by independent sample *t* test, and the experiment was repeated three times.

Pulmonary alveolar epithelial cells were regarded as the main source of inflammatory factors and chemokines. The immunofluorescence staining results of lung tissue sections of asthmatic mice showed that the surface markers of alveolar epithelial cells EPCAM and CDH1 and the chemokine CXCL10 were highly overlapped in the cells (Figure 5A), indicating that alveolar epithelial cells might be the main cells where CXCL10 was produced. The stimulative effect of ADP on asthma allergies may be caused by the infiltration of MCs, where CXCL10 plays an important role. In order to explore the molecular mechanism of ADP-mediated CXCL10 expression, alveolar epithelial cells SW1573 were treated with PSB0739, a specific inhibitor of P2Y1 receptor. The results showed that ADP-induced transcription and expression of CXCL10 could be inhibited by PSB0739 (Figures 5B,C). At the same time, the expression of the P2Y1 receptor in the alveolar epithelial cells SW1573 was silenced by siRNA, and both two siRNAs showed good knockdown efficiency (Figure 5D). It was also found that ADP-induced CXCL10 expression was significantly reduced at both the mRNA level and the protein level in P2Y1-knockdown cells (Figures 5E,F). Through the above experiments, ADP promoted CXCL10 expression through P2Y1 receptor, and hence may affect MC-mediated lung inflammation in asthma.

### ADP Promotes CXCL10 Secretion by Stimulating Alveolar Epithelial Cells

**FIGURE 6:**
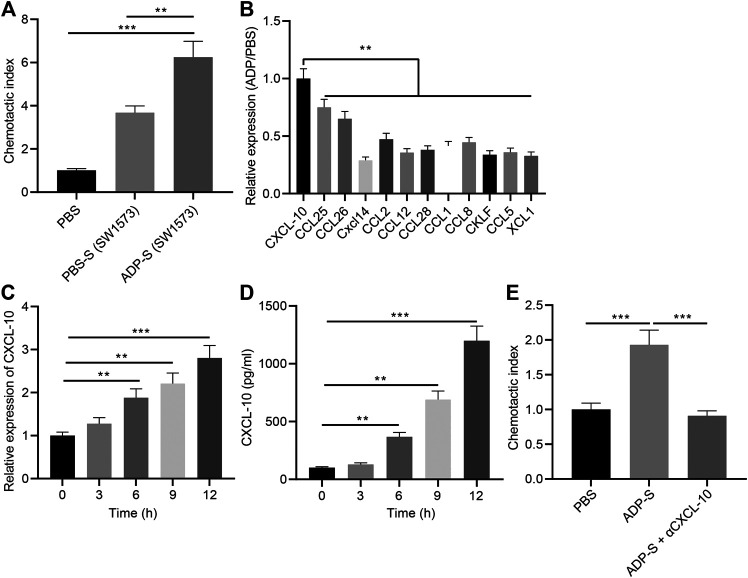
ADP promotes CXCL10 secretion by stimulating alveolar epithelial cells. **(A)** The migration index of HMC-1 incubated with ADP-stimulated cell culture supernatant assessed by Transwell assay. **(B)** The expression of various chemokines determined by RT-qPCR. **(C)** Quantitative analysis of the expression of CXCL10 in ADP-stimulated alveolar epithelial cells at different time points. **(D)** The level of CXCL10 in ADP-stimulated alveolar epithelial cells detected by ELISA at different time points. **(E)** The migration ability of MCs incubated with ADP-stimulated cell culture supernatant. *p < 0.05, **p < 0.01, and ***p < 0.001. Measurement data were expressed by the mean ± standard deviation, analyzed by independent sample *t* test, and the experiment was repeated three times.

In addition, we analyzed the mechanism by which extracellular ADP involved in airway inflammation. Human alveolar epithelial cells SW1573 were treated with ADP or control PBS for 12 h, and the culture supernatant was collected. Transwell chamber was used to detect the effect of ADP-stimulated culture supernatant of SW1573 cells on the migration ability of MC line HMC-1. The results found that the migration of MCs was significantly increased by the cell culture supernatant stimulated by ADP, thereby promoting their targeted recruitment (Figure 6A), which suggested that chemokines existed in the supernatant that could promote the migration of MCs. Therefore, the expression of a variety of chemokines in ADP-stimulated SW1573 cells was determined RT-qPCR, showing that the expression of CXCL10 was significantly higher than other chemokines. This was consistent with the elevated CXCL10 expression in the lungs of asthmatic mice that treated with ADP (Figure 6B). At the same time, the time-dependent promotion of CXCL10 transcription and protein expression in alveolar epithelial cells could be achieved by ADP stimulation (Figures 6C,D). On the contrary, after treatment with the CXCL10-neutralizing antibody in supernatant, the migration of MCs enhanced by the ADP-stimulated cell culture supernatant was suppressed (Figure 6E). The aforementioned results indicate that alveolar epithelial cells may be activated by ADP and release the chemokine CXCL10, which in turn recruit MCs to participate in aggravated asthma-induced lung inflammation.

### Extracellular ADP Activates NF-κB Signaling Pathway

**FIGURE 7:**
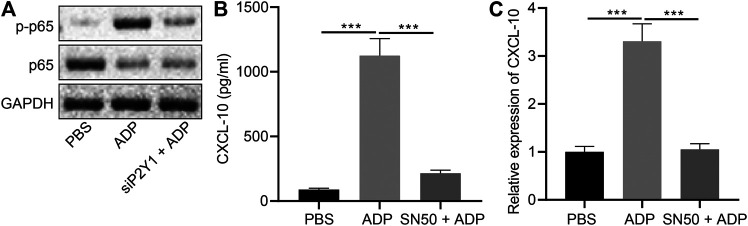
Extracellular ADP activates NF-κB signaling pathway. **(A)** Phosphorylation of p65 after P2Y1 knockdown or treatment with ADP. **(B)** release of CXCL10 in the SW1573 cells after SN50 treatment (1 h) and ADP treatment (12 h). **(C)** Quantitative analysis of the expression of CXCL10 in the SW1573 cells after SN50 treatment (1 h) and ADP treatment (12 h) by RT-qPCR. *p < 0.05, **p < 0.01, and ***p < 0.001. Measurement data were expressed by the mean ± standard deviation, analyzed by independent sample *t* test, and the experiment was repeated three times.

NF-κB signaling plays an important role in the ADP-P2Y1 signaling pathway (Suzuki et al., 2020). We observed nuclear translocation and phosphorylation of NF-κB p65 in alveolar epithelial cells after stimulation with ADP, which were reduced after P2Y1 knockdown (Figure 7A). Additionally, ADP-activated p65 phosphorylation was inhibited by P2Y1 knockdown (Figure 7B). When alveolar epithelial cells were treated with SN50, a specific inhibitor of NF-κB p65 phosphorylation, ADP-induced transcription and expression of CXCL10 was significantly inhibited (Figures 7C). Hence, extracellular ADP can activate the NF-κB signaling pathway, inducing nuclear translocation and phosphorylation of p65 to promote the expression of CXCL10, whereby participating in the asthma-induced lung inflammation.

## Discussion

A previous study has demonstrated that MCs increase in the airway during asthma followed by the infiltration of MCs in the epithelium, the submucosal glands, as well as the smooth muscle bundles (Elieh Ali Komi and Bjermer, 2019). Interestingly, the study of Yoshikawa et al*.* has suggested that CXCL10 stimulation can trigger the infiltration of Th1 cells into nasal polyps (Yoshikawa et al., 2013). A previous study has identified that ASMCs under the asthma conditions induce the migration of MCs through releasing adenosine triphosphate (ATP), the effect of which can be mimicked by ADP that produces results similar to or greater than those obtained using ATP (John et al., 2001; Pastore et al., 2007), and CXCL10 (Amison et al., 2015). Interestingly, P2Y1 and P2Y12 receptors can mediate ADP-induced platelet aggregation and hence therapeutic agents that selectively target P2Y1 and P2Y12 receptors are used for prevention and treatment of cardiovascular events (von Kugelgen and Hoffmann, 2016). Therefore, this study was designed to explore the potential role of extracellular ADP, an agonist of P2Y1 receptor in CXCL10-mediated MC infiltration and airway inflammation in asthma. The obtained results demonstrated that extracellular ADP stimulated the CXCL10-mediated MC infiltration through the P2Y1 receptor, thereby aggravating airway inflammation in asthma.

Initial experimental results revealed that the expression of extracellular ADP was relatively high in the BALF of patients with asthma and the expression of P2Y1 receptor was highly expressed in the lung tissues of asthmatic patients. Consistently, a previous study has also shown that the extracellular ATP level is increased in the BALF of patients with asthma (Chavez et al., 2019). Another study demonstrated that extracellular ADP can activate NLRP3 inflammasome to aggravate microglial inflammation in combination with ATP by acting on the P2Y12 receptor (Suzuki et al., 2020). However, the present study demonstrated that ADP in the BALF could deteriorate airway inflammation in asthma through P2Y1 receptor to recruit MCs. Additionally, after eliminating the endogenous ADP, the MC infiltration was reduced in OVA-sensitized asthmatic mice while administration of extracellular ADP could increase the number of infiltrated MCs, indicative of an increased airway inflammatory response. Strikingly, P2Y1 receptor but not P2Y12 antagonism represses recruitment of pulmonary leukocytes since P2Y1 activation-induced RhoA signaling is responsible for platelet-dependent leukocyte chemotaxis (Amison et al., 2015) which showed some similarities with our results that P2Y1 receptor was indispensable for the infiltration of MCs. Antagonists and inhibitors of platelet P2 receptors contribute to an anti-inflammatory role. In addition to platelet-related processes, P2Y1, P2Y12, and P2X1 receptors, expressed by cells of the immune system and by vascular cells, directly participate in the inflammatory and immune responses (Gachet and Hechler, 2020). Our experimental data showed that the asthma-related symptoms aggravated by ADP could be alleviated in P2Y1-knockout mice. Therefore, it could be concluded that extracellular ADP aggravated airway inflammation in asthma by activating the P2Y1 receptor.

Subsequently, the experimental results of the current study uncovered that the NF-κB signaling pathway was activated by extracellular ADP through the P2Y1 receptor, by which expression of CXCL10 was promoted, and MC infiltration and airway inflammation were thereby accelerated in asthma. Indeed, a recent work confirmed that extracellular ADP acts on the P2Y12 receptor to potentiate activation of the NF-κB signaling pathway during microglial inflammation (Suzuki et al., 2020). In accordance with this finding, the expression of CXCL10 has been observed to be reduced by specific antagonists of P2Y1 (Salem et al., 2018). Meanwhile, the mRNA expression and secretion of CXCL10 in ASMCs have been detected to be increased after stimulation with ATP (Gao et al., 2014). Furthermore, inhibition of NF-κB can reduce the release of CXCL10 which closely correlates with the migration of MCs to airway smooth muscle (Alrashdan et al., 2012; Gauthier et al., 2017). Consistent with our findings, a previous study has also shown that inhibition of the NF-κB signaling pathway through blocking its translocation represses the release of pro-inflammatory proteins (Ye et al., 2017). Notably, accumulating studies have identified that the airway inflammation is ascribed to the activation of the NF-κB signaling pathway (Girkin et al., 2020; Yang H. et al., 2020; Yang D. et al., 2021). Our study here demonstrated that inhibitor of NF-κB blocked the extracellular ADP-induced release of CXCL10 in alveolar epithelial cells. Taken together, the extracellular ADP augmented CXCL10-mediated MC infiltration through activating P2Y1 receptor/NF-κB, thereby aggravating the airway inflammation in asthma.

**FIGURE 8:**
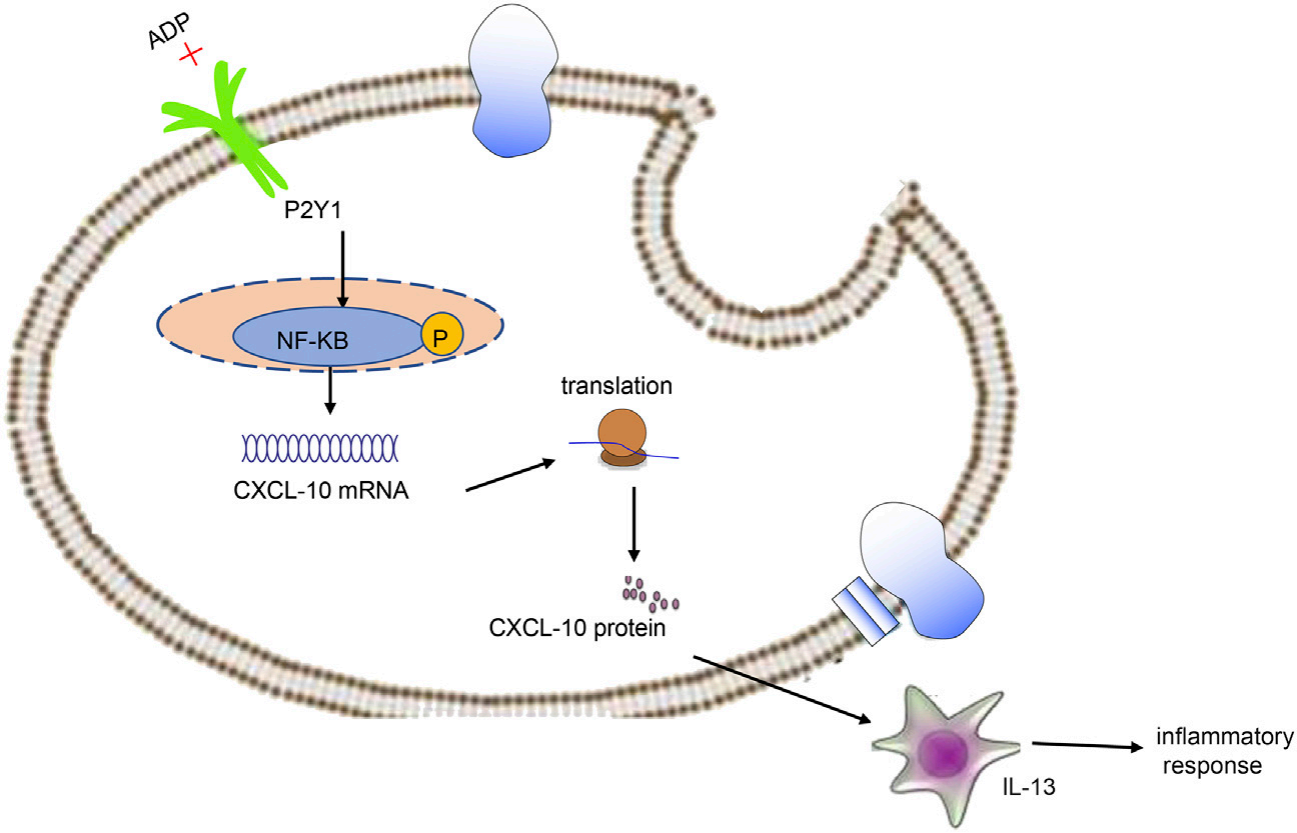
Extracellular ADP triggers and maintains airway inflammation in asthma by activating P2Y1 receptor and promoting the secretion of CXCL10.

In conclusion, high levels of extracellular ADP in asthmatic mice can augment CXCL10-evoked MC infiltration through P2Y1 receptor, thereby aggravating the airway inflammation in asthma (Figure 8). Thus, therapeutic strategies should be directed toward the down-regulation of extracellular ADP based on the evidence of this study, which may potentially be a promising anti-inflammatory target in the treatment of asthma. However, this study is still at the preclinical stage. In future studies, it is also recommended to validate the clinical application in a large sample size.

## Data Availability

The original contributions presented in the study are included in the article/Supplementary Material, further inquiries can be directed to the corresponding author.
